# miR-221/222 Promotes S-Phase Entry and Cellular Migration in Control of Basal-Like Breast Cancer

**DOI:** 10.3390/molecules19067122

**Published:** 2014-05-30

**Authors:** Yuan Li, Chunli Liang, Haizhong Ma, Qian Zhao, Ying Lu, Zhendong Xiang, Li Li, Jie Qin, Yihan Chen, William C. Cho, Richard G. Pestell, Li Liang, Zuoren Yu

**Affiliations:** 1 Research Center for Translational Medicine, Key Laboratory of Arrhythmias of the Ministry of Education, East Hospital, Tongji University School of Medicine, 150 Jimo Road, Shanghai 200120, China; E-Mails: liyuan_0806@hotmail.com (Y.L.); liang2006718@sina.com (C.L.); mhzygb@163.com (H.M.); tiankong74177@126.com (Q.Z.); luying_1010@hotmail.com (Y.L.); druid456@msn.com (Z.X.); flylily322@hotmail.com (L.L.); yihanchen@hotmail.com (Y.C.); 2 Lanzhou University School of Pharmacy, the First Affiliated Hospital of Lanzhou University, Lanzhou, Gansu 730000, China; E-Mail: liangli418@163.com; 3 Department of Anatomy, Histology and Embryology, Shanghai Medical College, Fudan University, Shanghai 200120, China; E-Mail: shhqj@shmu.edu.cn; 4 Department of Clinical Oncology, Queen Elizabeth Hospital, Hong Kong, China; E-Mail: williamcscho@gmail.com; 5 Department of Cancer Biology, Kimmel Cancer Center, Thomas Jefferson University, Philadelphia, PA 19107, USA; E-Mail: richard.pestell@jefferson.edu

**Keywords:** miR-221, miR-222, basal-like breast cancer, migration, cell cycle

## Abstract

The miR-221/222 cluster has been demonstrated to function as oncomiR in human cancers. miR-221/222 promotes epithelial-to-mesenchymal transition (EMT) and confers tamoxifen resistance in breast cancer. However, the effects and mechanisms by which miR-221/222 regulates breast cancer aggressiveness remain unclear. Here we detected a much higher expression of miR-221/222 in highly invasive basal-like breast cancer (BLBC) cells than that in non-invasive luminal cells. A microRNA dataset from breast cancer patients indicated an elevated expression of miR-221/222 in BLBC subtype. S-phase entry of the cell cycle was associated with the induction of miR-221/222 expression. miRNA inhibitors specially targeting miR-221 or miR-222 both significantly suppressed cellular migration, invasion and G_1_/S transition of the cell cycle in BLBC cell types. Proteomic analysis demonstrated the down-regulation of two tumor suppressor genes, suppressor of cytokine signaling 1 (SOCS1) and cyclin-dependent kinase inhibit 1B (CDKN1B), by miR-221/222. This is the first report to reveal miR-221/222 regulation of G_1_/S transition of the cell cycle. These findings demonstrate that miR-221/222 contribute to the aggressiveness in control of BLBC.

## 1. Introduction

Breast cancer is the most common non-cutaneous cancer in women. Although the traditional treatment therapies and surgery may decrease tumor size, reduce tumor growth rate and prolong patient survival, the 5-year survival in metastatic breast cancer is ~27% compared to 98% for localized breast cancer [1]. Metastatic breast cancer occurs in 20%–30% of women with breast cancer. The most common regions of breast cancer spread are to bone, lung, brain and liver. Metastatic breast cancer remains incurable due to the limited current understanding of the mechanisms governing metastasis [2].

microRNAs (miRNAs) are a class of non-coding small RNA which are processed from the original transcript, called primary miRNA (pri-miRNA), to precursor miRNA (pre-miRNA) by the internuclease Drosha/Pasha and its partner DGCR8. Following the export of pre-miRNA to the cell cytoplasm, pre-miRNA is cleaved by another internuclease Dicer to mature miRNA with ~20–22 nt in length. According to the newly released miRBase (Release 20: June 2013), around 2,000 mature miRNA molecules have been identified or predicted in human-origin cells and tissues. miRNA regulates target gene expression mostly through inhibiting translation but in some cases through cleavage of target gene mRNA via base-pairing with the 3' untranslated region (3'UTR) of target mRNAs. One miRNA may have hundreds of target genes, and one mRNA may contain multiple potential binding sites to different miRNAs. As such at least one third of human genes are predicted to be regulated by miRNAs [3]. miRNA function in a broad range of biological processes and pathways including embryonic development, stem cell self-renewal, differentiation, cancer onset, cancer progression and cancer metastasis [4,5,6].

miRNA involvement in breast cancer has been well demonstrated [7,8]. The high frequency of DNA copy number abnormality in miRNA-located regions throughout the human genome was reported based on the miRNA gene analysis on 55 human breast tumors and 18 human breast cancer cell lines [9]. The aberrant expression of mature miRNAs in human breast cancers was found by miRNA microarray and Northern blot analyses on 76 human breast tumor samples and 14 breast cell lines [10]. miR-21, the most abundant miRNA in human breast cancer MCF-7 cells, enhances MCF-7 cell proliferation *in vitro* and promotes MCF-7 derived tumorigenesis *in vivo* by inhibiting the expression of a subset of tumor suppressor genes including several p53-regulated genes [11]. miR-27 increases the human breast cancer MDA-MB-231 cell proliferation through regulating the cell cycle and cell division [12]. Our previous findings demonstrated the growth inhibitory function of miR-17/20 in MCF-7 cells by targeting *cyclin D1* [13], which is consistent with the transgenic studies in which miR-17 inhibited cellular growth and proliferation [14]. Overexpression of miR-205 and miR-200c inhibits TGF-β-induced EMT in breast cancer [15]. miR-335, miR-206, and miR-126 inhibit breast cancer metastasis and relapse [16]. miRNA let-7 inhibits self-renewal and induces differentiation of human breast cancer stem cells (CSC). Enforced expression of let-7 in human breast CSC inhibited cell proliferation and mammosphere formation *in vitro* and blocked tumorigenesis and tumor cell metastasis in animal models [17].

**Figure 1:**
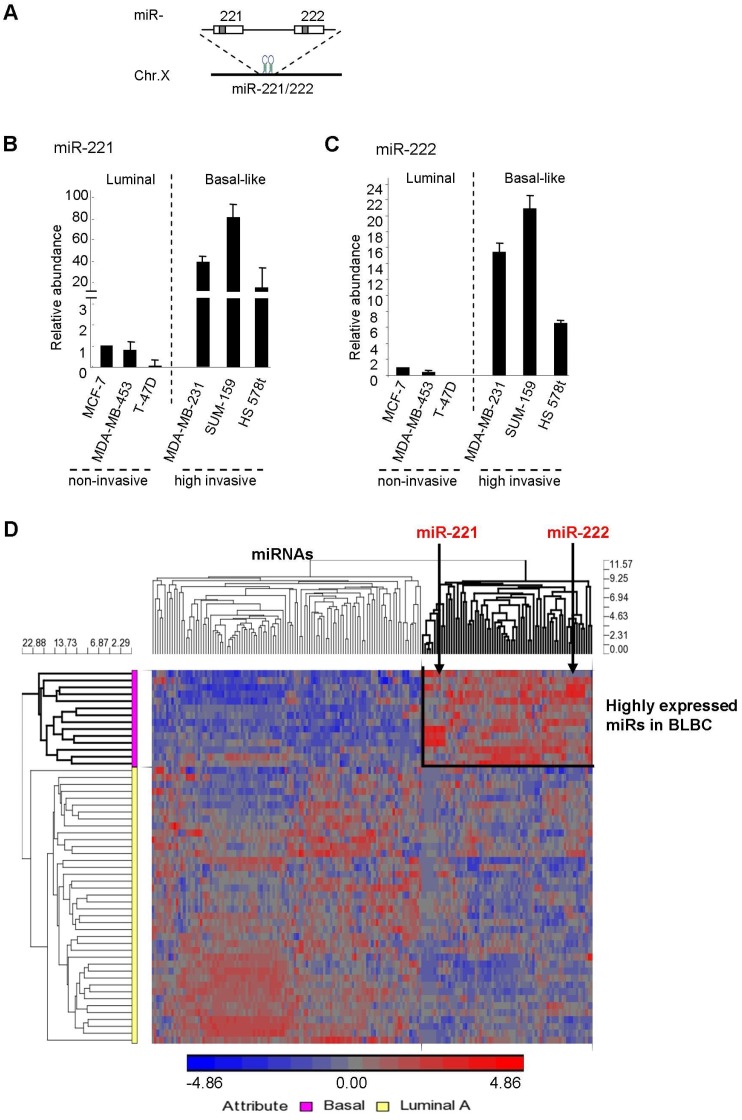
High expression of miR-221/222 in highly invasive BLBC. (**A**) Schematic indication of the miR-221/222 cluster on human chromosome X. (**B**, **C**) Relative abundance of miR-221 (**B**) and miR-222 (**C**) in highly invasive basal-like subtype (MDA-MB-231, Hs578t and SUM159) and non-invasive luminal subtype (MCF-7, T-47D and MDA-MB-453) breast cancer cell lines. Data are shown as mean ± SEM (SEM was derived from three independent experiments). (**D**) Tree view display of miRNA expression profile from 101 human breast cancer patient samples for luminal A and basal-like genetic subtypes. A subset of miRNAs including miR-221 and miR-222 showed higher expression in Basal than Luminal A subtype of breast cancer.

miR-221/222 is a miRNA cluster located on chromosome X (Figure 1A) where the genome abnormality occurs often contributing to the pathogenesis of basal-like human breast cancer [9,18]. Emerging evidence has demonstrated the regulation of breast cancer by miR-221/222 [19,20,21]. In addition, the miR-221/222 expression is associated with chemoresistance in breast cancer patients [22]. The regulation of miR-221/222 to the aggressive clinical behavior of BLBC [23,24] suggests a link between the expression of miR-221/222 and other oncogenes on chromosome X and aggressiveness of BLBC. Nevertheless, the mechanism by which the miR-221/222 cluster affects cellular proliferation, cell cycle, cellular migration and invasion in BLBC remains unclear. Here we demonstrate miR-221/222 promote cell migration and invasion in BLBC cell types. Induction of miR-221/222 expression was associated with G_1_/S transition of the cell cycle. miR-specific knockdown of miR-221/222 inhibited the cell cycle progression. In addition, we identified a novel target gene of miR-221/222, suppressor of cytokine signaling 1 (SOCS1), in human breast cancer.

## 2. Results and Discussion

### 2.1. High Expression of miR-221/222 in Highly Invasive BLBC

miR-221/222 regulates EMT [25,26]. In order to determine the effects of miR-221/222 on cellular migration and invasion in breast cancer, miR-221/222 expression was examined in highly invasive breast cancer cell lines Hs578t, MDA-MB-231 and SUM159, and in non-invasive breast cancer cell lines MCF-7, MDA-MB-453 and T-47D as well. Interestingly, miR-221 expression was ~20–80 times higher in invasive breast cancer cells than that in non-invasive cells (Figure 1B). Similarly, miR-222 expression was ~10–20 times higher in these invasive cells (Figure 1C).

Notably, all three of the MDA-MB-231, Hs578t and SUM159 cell lines belong to the BLBC genotype. In order to support above observation that mR-221 and miR-222 are much more abundant in invasive BLBC cells than non-invasive luminal cell types, a breast cancer miRNA-array dataset was compiled from the public repositories Gene Expression Omnibus (NCBI/GEO DATASETS:GSE19783) which was used to evaluate expression levels of miRNAs in 101 human clinical subtypes of breast cancer. A Higher expression of both miR-221 and miR-222 was found in BLBC patients compared to Luminal A subtype of breast cancer (Figure 1D and supplementary Figure S1), which is consistent with the observation from breast cancer cell lines.

### 2.2. miR-221/222 Promotes Cellular Migration and Invasion in BLBC

**Figure 2:**
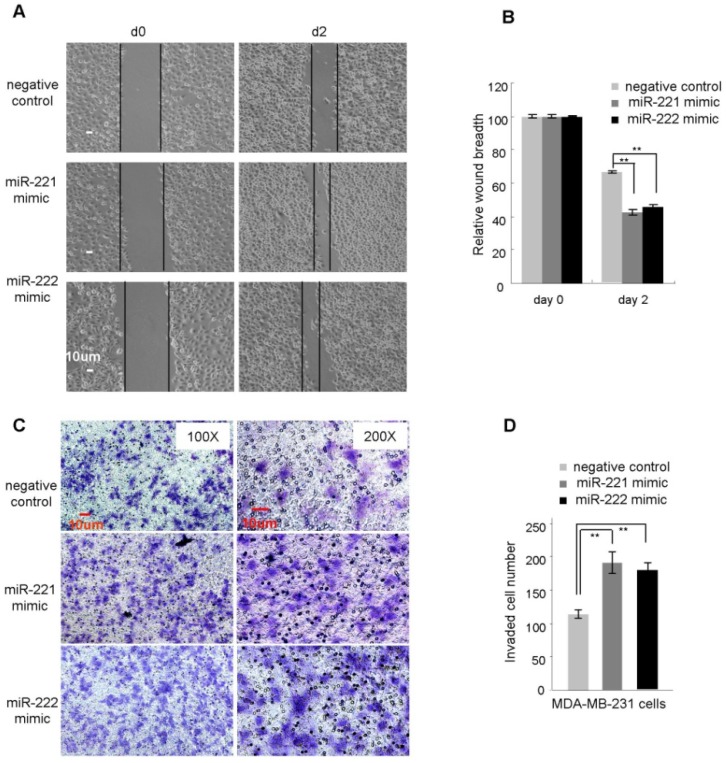
miR-221 and miR-222 promote cell migration and invasion in MDA-MB-231 cells. (**A**) Wound healing assays were performed on MDA-MB-231 cells (0.1% FBS) treated with miR-221 mimic, miR-222 mimic or negative control. The wound breadths were measured at the indicated time points. (**B**) Quantitative analysis of the cellular migration assayed in A. (**C**) Transwell invasion assay was performed on MDA-MB-231 cells treated with miR-221 mimic, miR-222 mimic or negative control. (D) Quantitative analysis of the invaded cells assayed in C. Data are mean ± SEM (*n* = 3). ** *p* < 0.01.

In view of the high expression of miR-221/222 in highly invasive BLBC cells, we examined the effect of miR-221/222 on migration and invasion of BLBC cell types. miR-221 and miR-222 mimics were transfected into MDA-MB-231 cells, respectively, followed by a wound healing migration assay and a transwell invasion assay. A scrambled RNA oligo was used as negative control. FBS-reduced cell-culture conditions (FBS concentration 0.1%–0.5%) were applied to minimize the effect from cell proliferation. As shown in Figure 2A,B, after 2-day incubation, the wounds were closed ~60% in miR-221 and miR-222 overexpressed conditions. By contrast, control cells showed ~30% wound closure. Transwell assays indicated the promotion of migration and invasion of MDA-MB-231 cells by enforced overexpression of miR-221/222 (Figure 2C,D). Similarly, miR-221 and miR-222 mimics were transfected into MCF-7 cells, no such effects on cellular migration were observed (data not shown).

**Figure 3:**
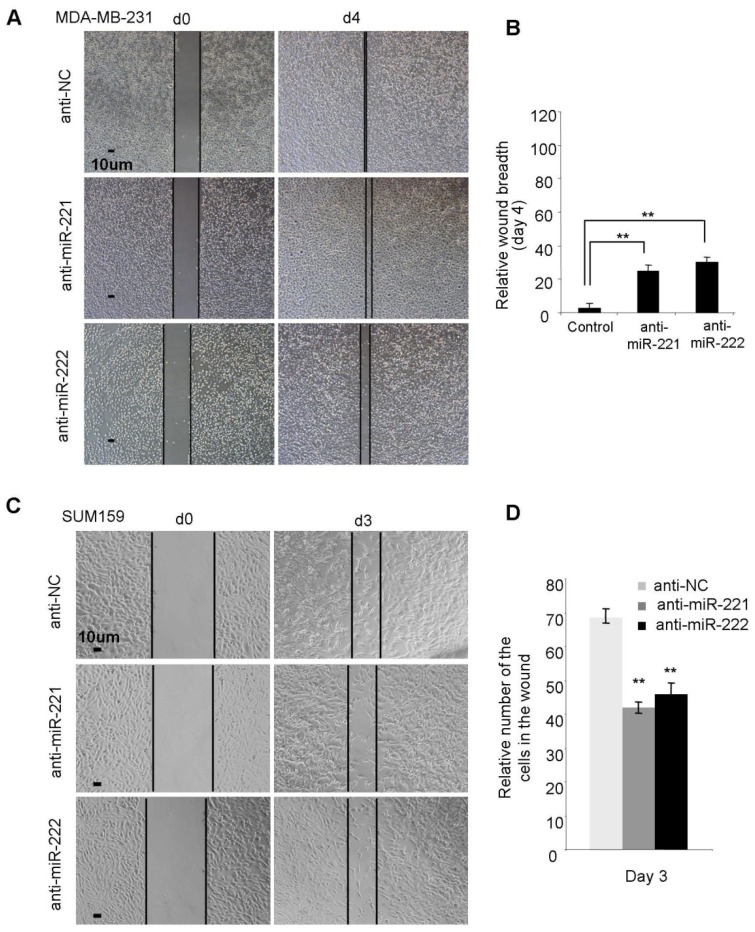
Anti-miR-221 and anti-miR-222 suppress cellular migration in BLBC cell types. (**A**) miRNA inhibitors targeting miR-221 and miR-222 both suppressed migration in MDA-MB-231 cells. (**B**) Quantitative analysis of the wound breadth at day 4 in A. (**C**) miRNA inhibitors targeting miR-221 and miR-222 both suppressed migration in SUM159 cells. (**D**) Quantitative analysis of the migrated cell numbers in the wounds at day 3 in C. Data are mean ± SEM (*n* = 3). ** *p* < 0.01.

To corroborate the effect of miR-221/222, anti-miR-221 and anti-miR-222 were applied to block the function of endogenous miR-221 and miR-222 in multiple BLBC cell lines including MDA-MB-231 (high expression of miR-221/222) (Figure 3A,B), SUM159 (high expression of miR-221/222) (Figure 3C,D) and Hs578t (moderate expression of miR-221/222) (Supplementary Figure S2) cells. The results demonstrated that inhibition of endogenous miR-221/222 is able to slow down wound closure in all the three BLBC cell types.

### 2.3. miR-221/222 Induce G_1_/S Transition of the Cell Cycle in BLBC

**Figure 4:**
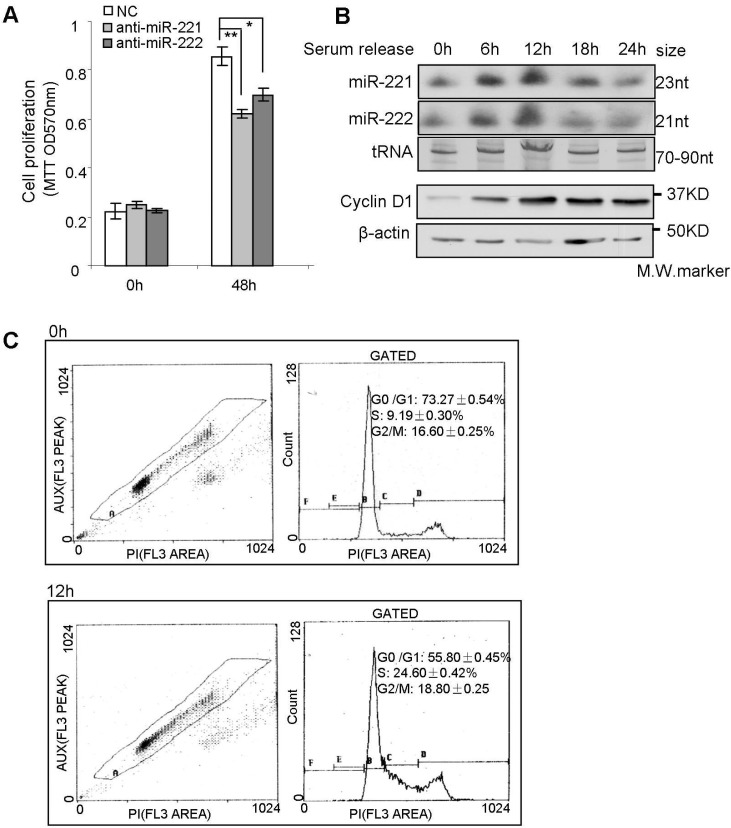
The induction of miR-221/222 expression is associated with S phase entry of the cell cycle. (**A**) MTT assays on MDA-MB-231 cells showing cell proliferation inhibition by both anti-miR-221 and anti-miR-222 after 48 h treatment. (**B**) Northern blot analysis on miR-221/222 expression in MEF cells at different stages of the cell cycle. tRNA served as RNA loading control; western blot analysis of cyclin D1 expression. β-actin served as protein loading control. (**C**) Cell cycle analysis demonstrated that serum-starved cells were arrested at G_0_/G_1_ phase (0h timepoint). G_1_/S transition occurred at 12 h timepoint after adding 10% FBS back. Values are equal to mean ± SEM (*n* = 4). * *p* < 0.05, ** *p* < 0.01

In order to determine the effects of miR-221/222 on the cell cycle and cell proliferation, MTT assays were performed, and demonstrated that miR-221/222 inhibitors could suppress cellular proliferation in BLBC MDA-MB-231 cells (Figure 4A). The expression of miR-221 and miR-222 was examined at different stages of the cell cycle. Serum-starved cells (0.5% FBS) showed low expression of both miR-221/222 which was associated with low cyclin D1 abundance (Figure 4B 0 h timepoint). After addition of 10% FBS for 12 h, both cyclin D1 and miR-221/222 expression were significantly induced (Figure 4B). Cell cycle analysis demonstrated that serum-starved cells were arrested at G_0_/G_1_ phase (G_1_:S = 73%:9%). After application with regular culture condition, G_1_/S transition and DNA synthesis occurred at 12h, where ~25% cells entered S phase (G_1_:S = 55%:25%) (Figure 4C and Supplementary Figure S3). The concurrence of miR-221/222 induction and G_1_/S transition suggested miR-221/222 may regulate the cell cycle.

**Figure 5:**
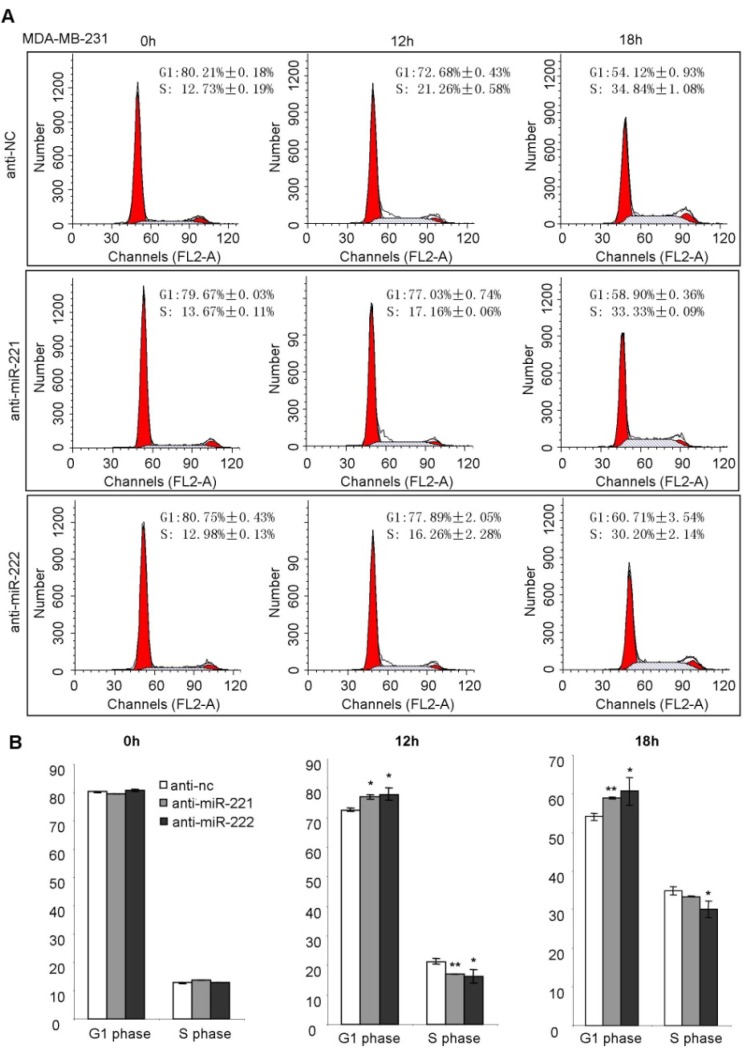
Anti-miR-221 and anti-miR-222 suppress G_1_/S transition of the cell cycle in MDA-MB-231 cells. (**A**) Cell cycle analysis on MDA-MB-231 cells at the starved status (0 h) and 10% FBS-released status (12 h and 18 h) showing inhibition of G_1_/S transition by miRNA both inhibitors targeting miR-221 and miR-222. (**B**) Quantitative analysis of the cell % at the G_1_ phase and S phase. Data are mean ± SEM (*n* = 3). * *p* < 0.05, ** *p* < 0.01.

In order to demonstrate the direct regulation of miR-221/222 on the cell cycle, miRNA inhibitors targeting endogenous miR-221 and miR-222 were applied to MDA-MB-231 cells, followed by a cell cycle analysis (Figure 5A). A quantitative analysis showed both anti-miR-221 and anti-miR-222 are able to inhibit the G_1_/S transition, and arrest the cell cycle at G_1_ phase (Figure 5A,B).

miRNAs are involved in tumorigenesis through regulating cell proliferation, and/or cell migration and invasion and tumor cell metastasis [4,27]. Stinson *et al* found that miR-221 and miR-222 promoted EMT in BLBC by inhibiting expression of epithelial-specific genes and increasing expression of mesenchymal-specific genes [28]. Croce’s group identified miR-221 as one of the nine miRNAs that differentiated invasive ductal carcinoma from ductal carcinoma *in situ* according to the miRNA analysis of >80 patient biopsies [29]. miR-221 was up-regulated in the invasive transition of ductal carcinoma. Consistent with the literature, our current finding demonstrated miR-221/222 promoted cellular migration and invasion in basal subtype of breast cancer.

### 2.4. miR-221/222 Inhibits the Expression of Tumor Suppressor Genes, SOCS1 and CDKN1B

**Figure 6:**
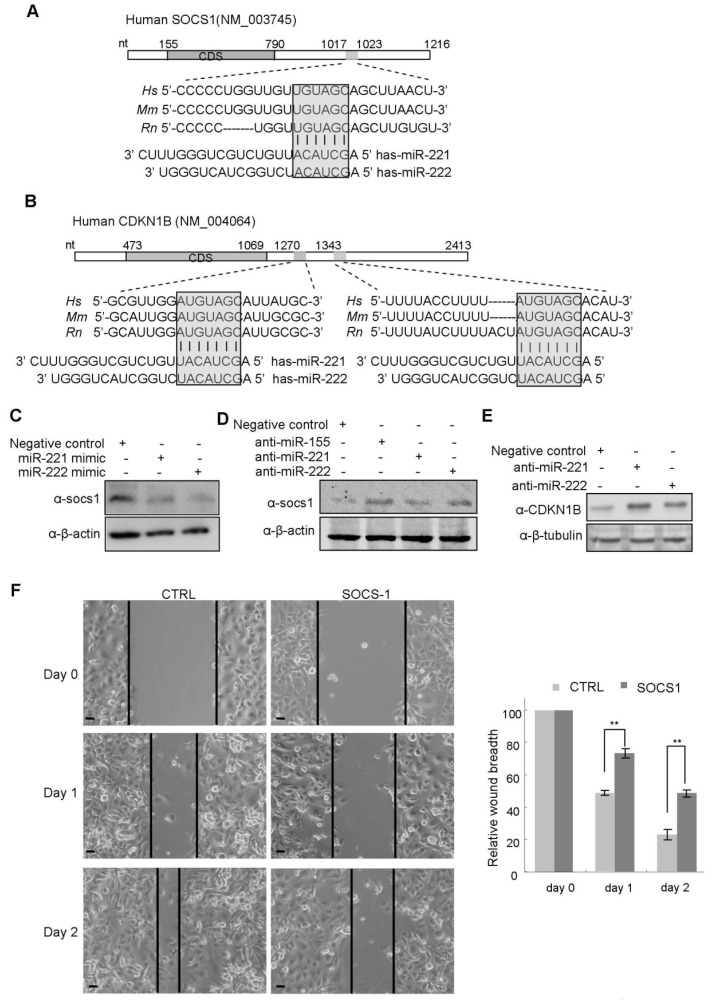
miR-221 and miR-222 suppressed expression of SOCS1 and CDKN1B. (**A**) Schematic representation of the Human SOCS1 3' UTR showing the highly conserved miR-221/222 binding site (highlighted and boxed) between species. The “seed” sequence of miR-221/222 (nt 2–7) is complementary to the SOCS1 3' UTR. (**B**) Sequence analysis of the 3' UTR of CDKN1B mRNA identified two conserved binding sites to the “seed” sequence of miR-221 and miR-222. (**C**) Western blot analysis indicating the inhibition of SOCS1 expression by miR-221/222 overexpression in MCF-7 cells. β-actin served as loading control. (**D**) Western blot analysis indicating the up-regulation of SOCS1 by miR-221/222 inhibitors in MDA-MB-231 cells. miR-155 was used as positive control for targeting SOCS1. β-actin served as loading control. (**E**) Western blot analysis demonstrating the up-regulation of CDKN1B by miR-221/222 inhibitors in MDA-MB-231 cells. β-tubulin served as loading control. (**F**) SOCS1 overexpression in MDA-MB-231 cells suppressed cellular migration. Data are mean ± SEM (*n* = 3). ** *p* < 0.01.

A sequence alignment analysis of mRNAs and miR-221/222 revealed one potential binding site in the 3'UTR of the suppressor of cytokine signaling 1 (SOCS1) gene (Figure 6A) and two binding sites in the 3'UTR of CDKN1B (Figure 6B), suggesting both SOCS1 and CDKN1B are candidates of miR-221/222 targets. Western blot analysis demonstrated the negative regulation of SOCS1 and CDKN1B expression by both miR-221 and miR-222 (Figure 6C–E) in breast cancer cells. Quantitative real time PCR analysis indicated the down-regulation of SOCS1 mRNA by miR-221/222 overexpression (Supplementary Figure S4). A luciferase reporter assay demonstrated the direct interaction between miR-221/222 and 3'UTR of CDKN1B (Supplementary Figure S5). Both miR-221 and miR-222 suppressed luciferase activity through interacting with CDKN1B 3'UTR. Targeted point mutation to the miR-221/222 binding site impaired such interaction. In comparison with miR-221 or miR-222, combination of miR-221 and miR-222 did not show more suppression to luciferase activity mainly due to the high conservation between the “seed” sequences of miR-221 and miR-222. It is very likely that either of the miRNA in cells may be saturated in interacting with CDKN1B and performing function.

In order to further demonstrate miR-221/222 function in breast cancer cells through targeting SOCS1 and CDKN1B, a plasmid carrying SOCS1 coding sequence was transfected into MDA-MB-231 cells in which SOCS1 has very low expression [30]. Compared to vector control, SOCS1 overexpression significantly suppressed cellular migration and wound closure in MDA-MB-231 cells (Figure 6F). In addition, Zou L. *et al.* reported that knock down of CDNK1B by siRNA promoted proliferation in MDA-MB-231 cells [31].

Taken together, these experimental evidences suggest miR-221/222 regulate the aggressiveness of basal-like subtype of breast cancer partly through inhibiting tumor suppressor genes SOCS1 and CDKN1B.

SOCS1 is a member of the suppressor of cytokine signaling family which negatively regulates cytokine signal transduction through inhibiting the Jak/Stat pathway [32]. SOCS1 expression is frequently lost in tumors including mammary gland tumor. The Stat3 signaling pathway regulates tumor metastases [33]. A recent publication reported that inactivation of Stat3 could suppress breast cancer brain metastases [34]. Our current work may reveal a novel mechanism through which miR-221/222 regulates migration, invasion and metastasis of breast cancer cells partly via downregulation of SOCS1 and activation of Stat3 signaling pathway.

The cyclin dependent kinase inhibitor CDKN1B is involved in the regulation of tumorigenesis. CDKN1B deficiency is associated with tumor progression and EMT induction in MMTV-Ras and MMTV-c-myc induced mammary gland tumors [35,36]. CDKN1B regulates the cell cycle as well. The inhibition of CDKN1B expression may be responsible for the promotion of cell proliferation by miR-221/222 in MDA-MB-231 cells.

## 3. Experimental

### 3.1. Cell Lines and Cell Culture

Human breast cancer cell lines, MDA-MB-231, MCF-7, MDA-MB-453 T-47D and Hs578t cells (ATCC) were cultured in DMEM medium containing penicillin and streptomycin (100 mg/L) and supplemented with 10% fetal bovine serum (FBS). SUM159 was cultured in Ham’s F-12 medium with 5% FBS, 5 μg/mL insulin, 1 μg/mL hydrocortisone and penicillin and streptomycin (100 mg/L).

### 3.2. Oligos and Transfection

Mimics and anti-miR inhibitors for miR-221, miR-222, miR-155 and corresponding negative controls were purchased from Ambion, Life Technologies (Austin, TX, USA). The Oligofectamine™ Transfection Reagent from Invitrogen, Life Technologies was used for cell transfection following the manufacturer’s instructions. Final concentration for miRNA mimics was 30 nM, for miRNA inhibitor was 50 nM.

### 3.3. miRNA Real Time RT-PCR Analysis

Total RNA was extracted with Trizol reagent (Invitrogen). First strand cDNA of miRNAs was prepared using the miRCURY LNA™ Universal cDNA Synthesis Kit (Exiqon, Vedbaek, Denmark) following the manufacturer’s instruction. Primer sets for real-time PCR of miR-221, miR-222 and 5S RNA were purchased from Exiqon. The SYBR Green Master Mix was ABI product (Applied Biosystem, Life Technologies). The ABI 7900 HT Sequence Detection System (Applied Biosystem) was used for quantitative real time PCR assay. 5S RNA was used for normalization.

### 3.4. Northern Blot Analysis

Northern blot analysis of miRNAs was performed as previously described [13]. Briefly, total RNA (10–20 µg/lane) was loaded on a 15% denaturing polyacrylamide gel and electophoresed at 200 V until the bromophenol blue approached the bottom. The RNA was transferred from the gel to Hybond-N^+^ membrane using a Semi-Dry Transfer Apparatus. DNA oligonucleotide probes (20–23 nt) were 5' end labeled with [γ-^32^P]-ATP and hybridization was carried out using Rapid-Hyb buffer following the manufacturer’s instructions (Amersham, Piscataway, NJ, USA).

### 3.5. Western Blot Analysis

Whole-cell lysates (50 μg) were prepared after 48h transfection of miRNA mimics or inhibitors, and separated by 10% SDS-PAGE, and the proteins were transferred to nitrocellulose membrane. After being blocked in 5% milk (w/v) at room temperature for one hour, the membranes were incubated at 4 °C overnight with primary antibodies (1:1,000). Following 1XPBST washing, the membranes were incubated with secondary antibodies (1:3,000) at room temperature for 1 h followed by ECL staining. The following antibodies were used for western blot: anti-SOCS1 from Abcam (ab62584); anti-cyclin D1 (sc-20044), anti-p27 (sc-776), anti-β-actin (sc-47778), and anti-β-tubulin (sc-9104) were purchased from Santa Cruz Biotechnology, Inc (Santa Cruz, CA, USA).

### 3.6. Wound Healing Assay

Cells were counted and plated in equal numbers in 12-well tissue culture plates to achieve 90% confluence. Thereafter, a vertical wound was created using a 0.1 µL pipette tip. The cells were cultured with FBS-reduced DMEM medium (0.1%–0.5% FBS). Images of the wound were captured at designated times to assess wound closure rate.

### 3.7. Cellular Invasion Assay

Around 2 × 10^4^ MDA-MB-231 cells were seeded on 8-μm-pore transwell filter insert (Corning Incorporated, Corning, NY, USA) coated with ECM Gel (E1270, Sigma-Aldrich, Saint-Louis, MO, USA).After 6 h incubation at 37 °C and 5% CO_2_, cells adherent to the upper surface of the filter were removed using a cotton applicator. Cells were stained with 0.4% violet crystal acetate overnight, and the numbers of cells invaded were counted.

### 3.8. Cell Proliferation Assays

For the 3-(4,5-dimethylthiazol-2-yl)-2,5-diphenyltetrazolium (MTT) assay, 4 × 10^3^ cells/well were seeded into 96-well plate in triplicate, after culturing for a period as indicated, the cells were stained with MTT solution for 3 h at cell-culturing condition followed by dissolving with DMSO. The cell growth was determined by measuring OD value at 570nm wavelength.

### 3.9. Cell Cycle Analysis

Cells were starved in DMEM supplemented with 5% charcoal-stripped serum or 0.5% regular FBS. After 24 h, medium was changed to DMEM with 10% normal FBS. Cells were harvested at different time points and cell cycle parameters were determined using laser scanning cytometry. Cells were processed by standard methods by using propidium iodide staining of cell DNA. 10,000 cells per sample were analyzed by flow cytometry with a FACScan flow cytometer (BD Biosciences, Mansfield, MA, USA).

### 3.10. Plasmid Transfection and Luciferase Reporter Assay

For cellular transfection with plasmid DNA, actively growing 293T cells were seeded on 12-well plates at a density of 1 × 10^5^ cells/well in an antibiotic-free medium. The next day, cells were transfected using lipofectamine 2000 (Invitrogen) with 1.0 μg of pGL3-p27 3'UTR and 0.2 μg of Renilla plasmid. Twenty-four hours after transfection, luciferase activities were measured using Dual-Luciferase Reporter Assay System by AutoLumat (Promega, Madison, WI, USA).

### 3.11. Statistical Analysis

Data are presented as mean ± SEM. The standard two-tailed Student’s t-test was used for statistical analysis, only *p* < 0.05 was considered significant.

## 4. Conclusions

We found an increased expression of miR-221/222 in basal-like subtype of human breast cancer. miR-221/222 promotes breast cancer cellular migration, invasion and S-phase entry of the cell cycle. We confirmed two tumor suppressor genes, suppressor of cytokine signaling 1 (SOCS1) and cyclin-dependent kinase inhibit 1B (CDKN1B), are negatively regulated in expression by both miR-221 and miR-222, which may have potential to be therapeutic targets for basal-like breast cancer treatment.

## Supplementary Materials

Supplementary materials can be accessed at: http://www.mdpi.com/1420-3049/19/6/7122/s1.
