# Supplementary material for: miR-221/222 Promotes S-Phase Entry and Cellular Migration in Control of Basal-Like Breast Cancer
Source: Molecules. 2014 May 30;19(6):7122–37. doi: 10.3390/molecules19067122 (PMC6271560; doi:10.3390/molecules19067122)

Supplementary Information

Figure S1. The list of miRNAs with elevated expression in BLBC patients.

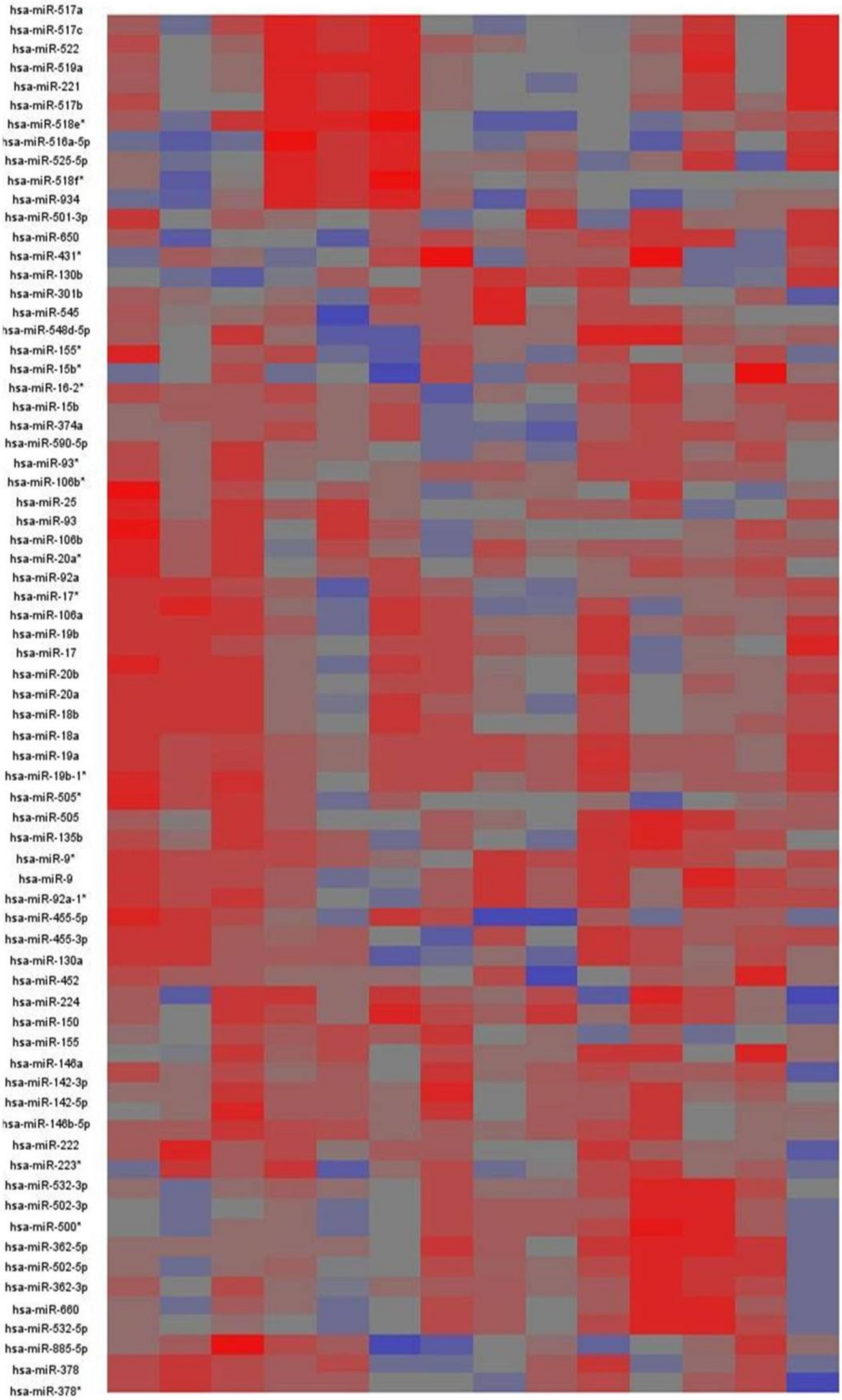

Figure S2. (**A**) miRNA inhibitors targeting miR-221 and miR-222 both suppressed migration in Hs578t cells. (**B**) Quantitative analysis of the migrated cell numbers in the wounds at day 3. Data are mean ± SEM ($n = 3$). ** $p < 0.01$.

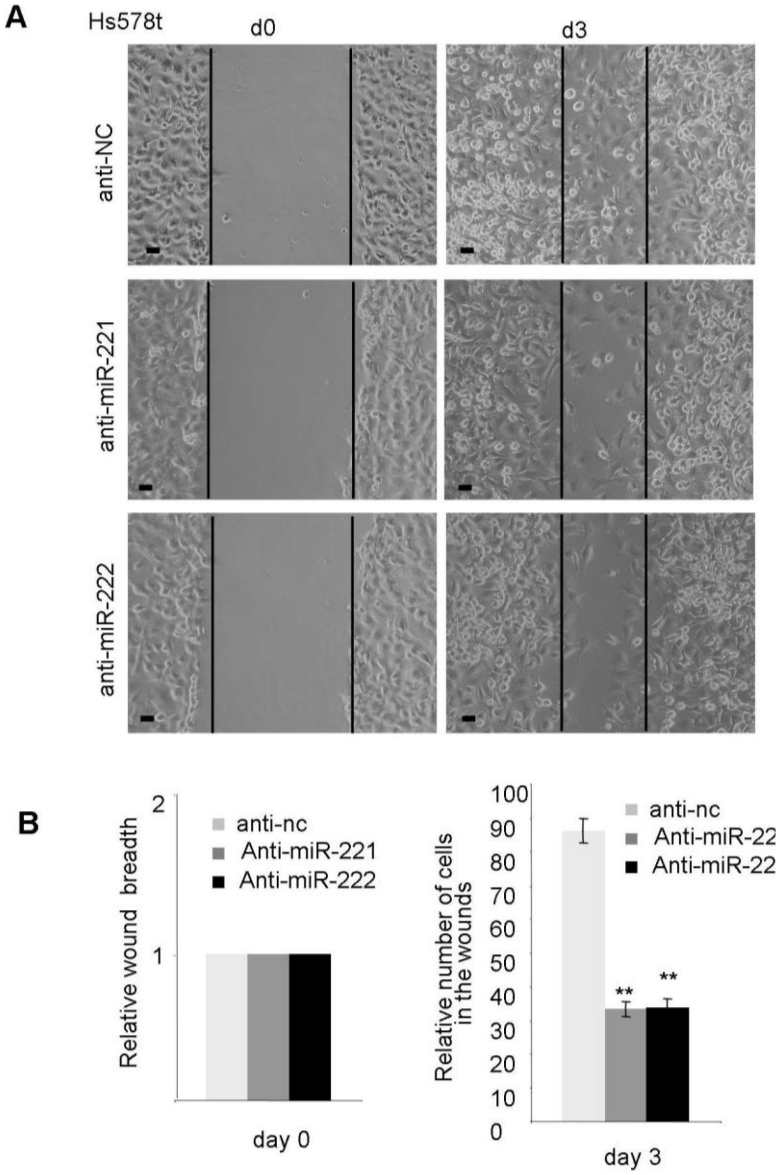

Figure S3. Quantitative analysis of the cell percentage at the different stages of a cell cycle in Figure 4C. * $p < 0.05$, ** $p < 0.01$.

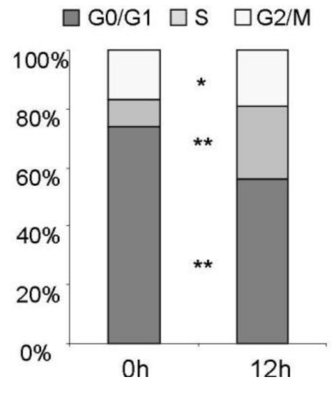

Figure S4. Quantitative real time PCR analysis showing SOCS1 mRNA decreased in miR-221- and miR-222- overexpressing MDA-MB-231 cells. Data are mean ± SEM ($n = 3$). ** $p < 0.01$.

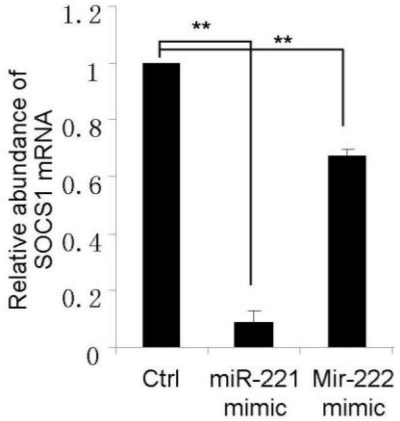

Figure S5. Luciferase reporter assays indicating direct interaction and inhibition of CDKN1B 3'UTR by miR-221 and miR-222. A mutated vector targeting miR-221/222 binding site did not show such inhibition by miR-221/222. Data are mean ± SEM ($n = 3$). * $p < 0.05$, ** $p < 0.01$.

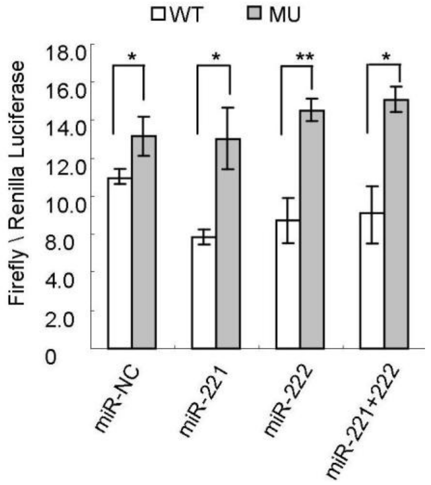

Supplement: Supplementary file 1 [file molecules-19-07122-s001.pdf]
